# Angioid streaks and optic disc drusen in cherubism: a case report

**DOI:** 10.5935/0004-2749.20200097

**Published:** 2024-02-11

**Authors:** Luiz Guilherme Marchesi Mello, Fábio Petersen Saraiva, Mario Luiz Ribeiro Monteiro

**Affiliations:** 1 Division of Ophthalmology, Faculdade de Medicina, Universidade de São Paulo, São Paulo, SP, Brazil; 2 Specialized Medicine Department, Universidade Federal do Espírito Santo, Vitória, ES, Brazil

**Keywords:** Angioid streaks, Optic disk drusen, Bruch membrane, Cherubism, Estrias angioides, Drusas do disco óptico, Lâmina basilar da cor**ó**ide, Querubismo

## Abstract

A 65-year-old female patient was referred to our hospital for evaluation for cataract surgery. Her past medical history included corrective jaw surgeries for facial deformities that developed during infancy and persisted through early adulthood. A complete ophthalmological examination revealed bilateral angioid streaks, drusen in both optic disc areas, and a subretinal neovascular membrane in the left macula. Genetic analysis revealed a mutation in the *SH3BP2* gene compatible with the diagnosis of cherubism. Clinical and laboratory evaluation revealed no additional systemic disorders. Cherubism is a rare disease characterized by the development of painless fibro-osseous lesions in the jaws and the maxilla in early childhood. Ophthalmologic findings in this disease are primarily related to orbital bone involvement. This is the first report of AS and optic disc drusen in a patient diagnosed with cherubism. Our findings suggest that angioid streaks and optic disk drusen should be included in the differential diagnosis of ophthalmic disorders identified in patients with this genetic abnormality.

## Abstract (pt)

Paciente de 65 anos, sexo feminino, foi encaminhada para avaliação de cirurgia de catarata. Relatou história de cirurgias mandibulares para correção de deformação facial desenvolvida ao longo da infância e adolescência. O exame oftalmológico completo mostrou estrias angióides bilaterais, drusas em ambas as áreas dos discos ópticos e membrana neovascular sub-retiniana na mácula esquerda. A análise genética revelou mutação no gene *SH3BP2* compatível com o diagnóstico de Querubismo. A avaliação clínica e laboratorial descartou outros distúrbios sistêmicos. O Querubismo é uma doença óssea rara caracterizada pelo desenvolvimento de lesões fibro-ósseas indolores na mandíbula e maxila durante a primeira infância. Os achados oftalmológicos nesta doença estão principalmente relacionados ao envolvimento ósseo orbitário. Este artigo descreve pela primeira vez a ocorrência de estrias angióides e drusas de disco óptico no Querubismo. Enfatizamos que essa condição deve ser incluída no diferencial de pacientes com tais achados, principalmente quando ambos existirem em associação.

## INTRODUCTION

Angioid streaks (AS) are peripapillary breaks in Bruch’s membrane (BM) that have been associated with systemic disorders^(1)^. AS is only rarely associated with optic disc drusen (ODD); the combination of these findings most often suggests the diagnosis of pseudoxanthoma elasticum (PXE), although it has also been described in disorders including Waldenstrom’s macroglobulinemia, β-thalassemia, and familial tumoral calcinosis^(1^,^2)^. Cherubism is a rare autosomal dominant disease caused by a mutation in the *SH3-domain binding protein 2* (*SH3BP2*) gene. Patients diagnosed with cherubism develop painless multilocular cysts in the jaws and the maxilla in early childhood which resemble the chubby cheeks of the putti (often confused with cherubs) in Renaissance paintings. This condition can undergo spontaneous regression after puberty^(3)^. Proptosis, strabismus, globe displacement, nasolacrimal duct obstruction, lower lid retraction and optic nerve impairment are among the common ophthalmologic manifestations of cherubism^(4)^. Patients may also present with abnormal retinal findings, including macular scarring, chorioretinal folds, inner retinal striae, retinoschisis and foveal vitelliform lesions^(5)^. To the best of our knowledge, this is the first report that describes both AS and ODD in a patient with cherubism.

## CASE REPORT

**Figure 1:**
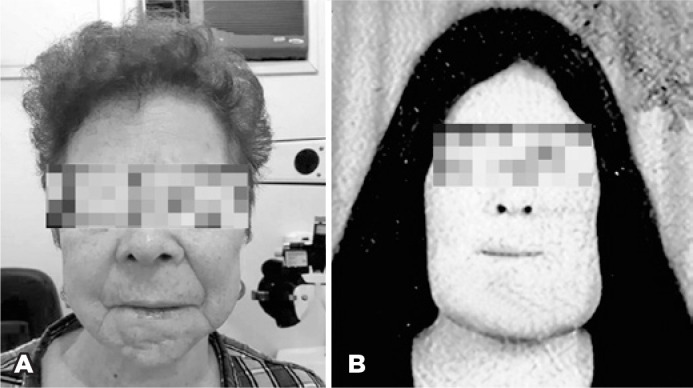
Photographs showing frontal view of the patient’s face. A) At presentation to our service at 65-years-old; B) In early adulthood, prior to surgeries for repair of the jaw deformity.

A 65-year-old female patient was referred for evaluation for cataract surgery. While her facial appearance was unremarkable at presentation (Figure 1A), her past medical history was notable for facial deformation from infancy through early adulthood (Figure 1B) that ultimately required several corrective jaw surgeries.

**Figure 2:**
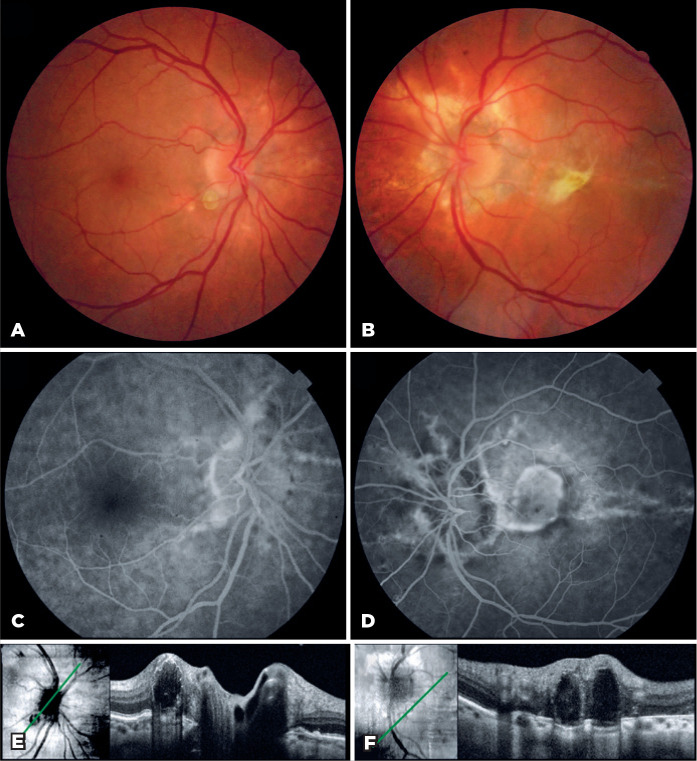
Fundus photograph shows irregular radial peripapillary lines and inferotemporal optic disc drusen in both eyes (A, B), and a yellowish subretinal lesion in the left eye (B). Fluorescein angiogram was notable for hyperfluorescence of the peripapillary crack-lines in both eyes (C, D), and a subretinal neovascular membrane in the left eye (D). Optical coherence tomography scan of both eyes demonstrates hyporeflective ovoid peripapillary structures surrounded by hyperreflective curvilinear bands, corresponding to optic disc drusen (E, F).

On examination, her best-corrected visual acuity was 20/70 in the right eye (OD) and counting fingers at 30 cm in the left eye (OS). Fundoscopy revealed bilateral irregular gray lines radiating from the peripapillary area, yellowish pearl-like nodulations in both optic discs, and an elevated yellow subretinal macular lesion in OS (Figure 2 A-B). Fluorescein angiography demonstrated bilateral centrifugal hyperfluorescent radial peripapillary crack-lines, a finding indicative of AS, and choroidal neovascularization in the left macula (Figure 2 C-D). Optical coherence tomography showed optic disc and peripapillary hyporeflective ovoid images bordered by hyperreflective bands compatible with a diagnosis of ODD (Figure 2 E-F).

Genetic analysis revealed a heterozygous missense mutation in the gene encoding *SH3BP2*, c.1253C>G (p.Pro418Arg), indicating a diagnosis of cherubism. Clinical and laboratory findings revealed no additional systemic disorders. Of particular note, a thorough dermatological examination and normal *ABCC6* gene ruled-out the diagnosis of PXE.

## DISCUSSION

SH3BP2 has been implicated in immune signaling and osteoclast differentiation, notably bone remodeling^(3)^. Wang et al., showed that an underlying systemic inflammatory process led to abnormal matrix deposition and altered collagen cross-linking in an animal model of cherubism^(6)^. The early stages of the disease feature osteo lytic activity in association with multiple multinucleated giant osteoclast-like cells. Subsequently, an increase in proliferative spindle cells, fibroblastic nodules, newly formed bone matrix, and osteoid are observed. In the final stages, new bone is generated in the presence of osteoblasts and mineralizing matrix^(3)^. Ophthalmologic findings in cherubism have primarily focused on orbital bone involvement, which may lead to a displacement of the globe and/or extraocular muscles. Genetic testing is essential to confirm the diagnosis due to its clinical, radiological, and histological similarity to other bone diseases^(3)^.

To the best of our knowledge, this is the first description of AS and ODD in a patient with cherubism. Mechanical stress and dehiscence of abnormally rigid collagenous and elastic layers of the calcified BM is thought to be a critical feature contributing to the pathogenesis of AS^(1)^. ODD are extracellular calcified deposits of mucoprotein matrix secondary to mitochondrial extrusion and chronic disturbance in optic nerve fiber metabolism^(7)^. While the mechanisms underlying disease pathogenesis are not known, it is possible that an imbalance between proand anti-mineralization factors may lead to abnormal calcium metabolism with calcification of the collagen and elastic fibers in the BM resulting in AS as well as hyaline bodies in optic nerve head and peripapillary retina in cherubism.

In conclusion, our case documents both AS and ODD in a patient with cherubism. Our findings suggest that AS and ODD should be included in the differential diagnosis of ophthalmic concerns among patients with this genetic abnormality.
